# Predictors of positive blood culture and deaths among neonates with suspected neonatal sepsis in a tertiary hospital, Mwanza- Tanzania

**DOI:** 10.1186/1471-2431-10-39

**Published:** 2010-06-04

**Authors:** Neema Kayange, Erasmus Kamugisha, Damas L Mwizamholya, Seni Jeremiah, Stephen E Mshana

**Affiliations:** 1 Department of Pediatric and Child Health Bugando Medical Centre, Mwanza, Tanzania; 2 Department of Biochemistry Weill Bugando University College of Health Sciences, Mwanza, Tanzania; 3 Department of Microbiology/Immunology Weill Bugando University College of Health Sciences Mwanza, Tanzania

## Abstract

**Background:**

Neonatal sepsis is a significant cause of morbidity and mortality in neonates. Appropriate clinical diagnosis and empirical treatment in a given setting is crucial as pathogens of bacterial sepsis and antibiotic sensitivity pattern can considerably vary in different settings. This study was conducted at Bugando Medical Centre (BMC), Tanzania to determine the prevalence of neonatal sepsis, predictors of positive blood culture, deaths and antimicrobial susceptibility, thus providing essential information to formulate a policy for management of neonatal sepsis.

**Methods:**

This was a prospective cross sectional study involving 300 neonates admitted at BMC neonatal unit between March and November 2009. Standard data collection form was used to collect all demographic data and clinical characteristics of neonates. Blood culture was done on Brain Heart Infusion broth followed by identification of isolates using conventional methods and testing for their susceptibility to antimicrobial agents using the disc diffusion method.

**Results:**

Among 770 neonates admitted during the study period; 300 (38.9%) neonates were diagnosed to have neonatal sepsis by WHO criteria. Of 300 neonates with clinical neonatal sepsis 121(40%) and 179(60%) had early and late onset sepsis respectively. Positive blood culture was found in 57 (47.1%) and 92 (51.4%) among neonates with early and late onset neonatal sepsis respectively (p = 0.466). Predictors of positive blood culture in both early and late onset neonatal sepsis were inability to feed, lethargy, cyanosis, meconium stained liquor, premature rupture of the membrane and convulsion. About 49% of gram negatives isolates were resistant to third generation cephalosporins and 28% of *Staphylococcus aureus *were found to be Methicillin resistant *Staphylococcus aureus *(MRSA). Deaths occurred in 57 (19%) of neonates. Factors that predicted deaths were positive blood culture (p = 0.0001), gram negative sepsis (p = 0.0001) and infection with ESBL (p = 0.008) or MRSA (p = 0.008) isolates.

**Conclusion:**

Our findings suggest that lethargy, convulsion, inability to feed, cyanosis, PROM and meconium stained liquor are significantly associated with positive blood culture in both early and late onset disease. Mortality and morbidity on neonatal sepsis is high at our setting and is significantly contributed by positive blood culture with multi-resistant gram negative bacteria.

## Background

World Health Organization (WHO) estimates about 5 million neonatal deaths a year. Almost all deaths occur in developing countries; half of them in the African region [1]. Septicemia is a common cause of infants' morbidity and mortality in developing countries [1]. There is no pathognmonic features of neonatal sepsis [2]; clinical presentation of neonatal sepsis can vary; in most studies symptoms include fever, difficult in breathing, tachycardia, malaise, difficult in feeding and lethargy [1,3,4]. It is therefore important to carry out investigation to confirm neonatal sepsis in the setting of good laboratory facilities and services. In the study done in Kenya, difficult in feeding, unexplained pallor, cyanosis and unconsciousness were strongly associated with severe sepsis while fast breathing, nasal flaring, grunting and lethargy were found to be associated with moderate form of sepsis [5]. WHO has made criteria for initial diagnosis of neonatal sepsis, but the sensitivity and specificity of clinical diagnosis can vary considerably [1,2,6].

Prompt diagnosis and effective treatment is necessary to prevent deaths and complications due to septicemia. Physical signs and symptoms are useful in identifying infants and children with septicemia. These clinical characteristics can be good predictors for positive blood culture but they have limited specificity and sensitivity [7. 8]. Neonatal blood culture positive rate have been found to range from 25-54% [5,9-11]. Rapid immunological techniques like C-Reactive Proteins (CRP) assays may help in the diagnosis of septicemia; however they lack the capacity to detect specific pathogens and are not available in many centers in developing countries [12]. Blood culture to isolate the offending pathogen remains the gold standard for definitive diagnosis of septicemia [1,12] but the results of blood culture takes hours to days, thus necessitating initial empirical treatment of suspected cases. Knowledge of predictors of positive blood culture and anti-microbial susceptibility pattern of common pathogens in a given area is essential in guiding local empirical choice of antibiotics [12].

At Bugando Medical Centre (BMC) neonatal units' ampicillin and gentamicin are used as the first line empirical treatment, the decision of this regimen is based on research in different units in developed countries. The antimicrobial susceptibility may vary between units and there is increasing resistance rate of gram negative bacteria to ampicillin gentamicin and cefotaxime worldwide; this warrants the availability of local data [13].

In Tanzania, as in other Sub-Saharan countries the epidemiology of neonatal sepsis has not been extensively studied and therefore to date most of empirical treatments have been formulated using data from developed countries [1,5]. Thus this study was done to determine the pattern of neonatal sepsis and the outcome at BMC, Mwanza Tanzania in order to generate local data that will be used for local policy formulation in managing neonatal sepsis. The predictors of positive blood culture and deaths established in the present study are useful to Pediatricians locally and in other centers for aggressive management of neonatal sepsis.

## Methods

### Study design and setting

This was a hospital based prospective cross-sectional study conducted at Bugando Medical centre (BMC) neonatal unit from March to November 2009. BMC is a tertiary facility which handles referrals from peripherals in the Lake zone of Tanzania; it serves 13 million Tanzanians.

### Study population

Sample size was calculated using Kish and Lisle methods, using P of 20% about 300 neonates were targeted [14]. During the study period 770 neonates were admitted in the unit and assessed using WHO sepsis screening tool and 300 neonates with clinical neonatal sepsis and had a blood culture drawn were included in the analysis [6]. These neonates included those delivered at BMC and referral from other centers in the Lake Zone.

### Data collection

Using guidelines laid down by WHO young Infant Study Group [6] a standard structured data collection form was designed to obtain social demographic data and other relevant information such as maternal fever, premature rupture of membranes (PROM) weight of the baby, gestational age, temperature of the infant, respiration rate, mode of delivery, presence or absence of cyanosis, jaundice, umbilical redness, convulsion, reduced movement and ability to feed. Appropriate treatment was initiated after collection of blood and neonates were followed until discharge or death; hospital stay to discharge or deaths was recorded in days.

### Blood culture

Using aseptic technique about 2 ml-5 ml of blood was obtained and inoculated directly into Brain Heart Infusion broth (BHI) (Oxoid Ltd, UK) in a ratio of blood: BHI of 1:10 and transported to the WBUCHS Microbiology laboratory for incubation and subsequent processing.

### Lab procedures

Aerobic Blood culture was done; after 24 hrs incubation gram stain was done followed by blind subculture on 5% sheep blood agar, chocolate agar, MacConkey agar and MacConkey with 30μg/ml cefotaxime (Oxoid Ltd, UK). Broth cultures were further re-incubated and then sub cultured after 48 hours then after 96 hrs with last subculture on day 7. Identification of bacteria was made by conventional physiological and biochemical methods. These included gram stain, catalase reaction, coagulase reaction, hemolytic activity on sheep blood agar plates, hippurate hydrolysis and CAMP tests for gram positive bacteria. In case of gram negative colonies morphology on blood and MacConkey agar, triple sugar iron agar reaction, indole, motility, citrate, urease, hydrogen sulphide production and VP test were used [15].

Finally, antimicrobial susceptibility of all isolates was determined by disk diffusion method according to Clinical Laboratory Standard Institute [16]. For gram positive bacteria discs tested included; penicillin G (10 U), ampicillin (10 μg), clindamycin (2 μg), erythromycin (15 μg), vancomycin (30 μg), ciprofloxacin (5 μg), oxacillin (5 μg) and cefoxitin (30 μg). For gram negatives discs used were ampicillin (10 μg), amoxyillin/clavunate (20/10 μg), ciprofloxacin (5 μg), tetracycline (30 μg), gentamicin (10 μg), Sulphamethaxazole/trimethoprim (SXT)1.25/23.75 μg) and ceftriaxone (30 μg). Other reserve discs included ceftazidime (30 μg), cefepime (30 μg) and meropenem (10 μg) (Oxoid UK). Isolates were screened for ESBL production using MacConkey agar with 30 μg /ml cefotaxime and confirmed using disc approximation method [15,16].

### Data analysis

The early onset sepsis was defined as disease occurring in ≤72 hours of age and late onset sepsis that occurring more than 72 hours of age [7]. Separate multivariable analyses were carried out to identify risk factors associated with early and late onset sepsis. Organisms causing, and outcomes of cases with early and late onset sepsis were also compared. Data was analyzed using SPSS for windows version 11.0 [17]. Statistical test between dependent and independent variables was done using Chi-squared test (χ2). Where the numbers in a cell was less than five, a Fisher's exact test was used. P-values ≤ 0.05 were considered statistically significant. All variables with association using univariate analysis were subjected to multivariate analysis.

### Ethical issues

Ethical clearance was obtained from BMC/WBUCHS ethical review board and a written informed consent was obtained from each mother/caretaker.

## Results

### Characteristic of study population

Three hundred neonates with clinical sepsis were enrolled in the study between March and November 2009. This was about 39% of neonates admitted during the study period. Early and late neonatal sepsis occurred in 121(40%) and 179(60%) respectively (Table 1).

**Table 1:** Background characteristics and positive blood culture among neonates with early and late onset neonatal sepsis

Parameter	Early onset sepsis ≤ 72 hrs			Late onset sepsis > 72 hrs		
	**N**	**Culture** **positive** **n(%)**	**P = Value**	**N**	**Culture positive** **n(%)**	**P = Value**

**Sex**						
Female (161)	63	30 (47.6)		98	45 (45.9)	
Male (139)	58	27 (46.6)	0.906	81	47 (58.0	0.107

**Mode of delivery**						
C/S (75)	31	12 (38.7)		44	24 (55.8)	
SVD (225)	90	45 (50.0)	0.277	135	68 (50.0)	0.313

**Gestation age**						
28-32 (22)	10	7 (70.0)		12	8 (66.7)	
33-36 (93)	34	14 (41.2)		59	34 (57.6)	
34-41 (185)	77	36 (46.8)	0.274	108	50 (46.3)	0.206

**Delivery place**						
Home (114)	38	15 (39.5)		76	30 (39.5)	
Hospital (186)	83	42 (50.6)	0.255	103	62 (60.2)	0.006

**Maternal fever**						
Yes (70)	31	17 (54.8)		39	26 (66.7)	
No (230)	90	40 (44.4)	0.317	140	66 (47.1)	0.031

**Meconium stain**						
Yes (91)	42	34 (81.0)		49	42 (85.7)	
No (209)	79	23 (29.1)	0.0001	130	50 (38.5)	0.0001

**PROM**						
Yes (89)	41	33 (80.5)		48	41 (85.4)	
No (211)	80	24 (30.0)	0.0001	131	51 (38.9)	0.0001

**Birth weight**						
1- 2.5 kg (97)	42	20 (47.6)		55	34 (61.8)	
2.6 - 5 kg (203)	79	37 (46.8)	0.7	124	59 (46.8)	0.045

### Culture results and susceptibility pattern

Of 300 neonates with clinical sepsis positive blood culture was found in 57(47%) and 92 (51.4%) among neonates with early and late neonatal sepsis respectively. Gram negative bacteria 91 (61.1%) were more frequently isolated than gram positive bacteria. Common isolates were *Klebsiella pneumoniae*, *Staphylococcus aureus *and *Escherichia coli*. Other pathogens were Coagulase negative staphylococcus (CNS) *Acinetobacter spp*, *Enterobacter spp *and other gram positive bacteria (*Listeria spp*, Group B streptococci and *Enterococcus spp*) (Table 2). *Escherichia coli*, *Klebsiella pneumoniae*, *Enterobacter spp *and Group B streptococci were commonly recovered in neonates from mothers with PROM. Eighty two percent of *Klebsiella pneumoniae *and 76% of *Escherichia coli *were isolated from neonates delivered in hospital.

**Table 2:** Isolates and resistance pattern of 149 isolates recovered from blood of 300 neonates

Drugs	*K. pneumoniae (50)*	*E. coli(22)*	*Other GNB(15)	*S. aureus (32)*	*CNS(14)*	*Other GPB(16)
Ampicillin	100%	100%	93%	NT	NT	50%
AMC	84%	86%	93%	NT	NT	NT
SXT	79%	77%	80%	60%	60%	NT
Tetracycline	62%	59%	93%	NT	NT	NT
Gentamicin	67%	68%	66%	NT	NT	68%
Ceftriaxone	50%	50%	53%	NT	NT	NT
Cefotaxime	49%	50%	53%	NT	NT	NT
Ceftazidime	49%	50%	46%	NT	NT	NT
Ciprofloxacin	8.2%	4.5%	13%	14%	17%	43%
Meropenem	2%	4.5%	0%	NT	NT	NT
Vancomycin	NA	NA	NA	14%	0%	0%
Erythromycin	NA	NA	NA	66%	50%	75%
Penicillin	NA	NA	NA	91%	86%	50%
Clindamycin	NA	NA	NA	44%	29%	56%
Cloxacillin	NA	NA	NA	28%	43%	62.5%

GNB: Gram negative bacteria, GPB: Gram positive bacteria, NA: Not applicable, NT: Note tested, SXT: Sulphamethaxazole/trimethoprim, AMC: Amoxicillin/Clavulanic acid

Most of *Klebsiella pneumoniae *and *Escherichia coli *were resistant to ampicillin and gentamicin with high proportion of gram positive bacteria being resistant to cloxacillin (first line treatment in the unit) (Table 2). More than 49% of *Klebsiella pneumoniae, Escherichia coli *and other gram negative bacteria were resistant to third generation cephalosporins (ceftriaxone, cefotaxime and ceftazidime) with majority of them being ESBL producer. Majority of these gram negative isolates were sensitive to ciprofloxacin and meropenem. Among 32 *Staphylococcus aureus *9 (28%) were found to be Methicillin Resistant *Staphylococcus aureus *(MRSA) (i.e resistant to oxacillin and cefoxitin) (Table 2).

### Factors associated with positive blood culture and increased mortality in early and late neonatal sepsis

No significant difference in positive blood culture results was observed between early and late onset sepsis. Percentage of neonates with early onset sepsis born at home was 31% and that of neonates with late onset sepsis born at home 42%. Among neonates with late onset sepsis those delivered in hospital had higher positive blood culture rate than those delivered at home (p = 0.006) (Table 1). Perinatal factors like PROM and meconium stained liquor were strongly associated with positive blood culture in both early and late onset sepsis (p ≤ 0.001). Gestational age and small gestational age by birthweight were not found to influence rate of positive blood culture in both early and late onset neonatal sepsis (Table 1). Clinical characteristics which were found to be significantly associated with positive blood culture on both univariate and multivariate analysis in early and late onset sepsis were inability to feed, lethargy, convulsion and cyanosis (Table 3). Increase in respiratory rate was only found to be a predictor of positive blood culture in early onset sepsis whereas hypothermia, chest indrawing, umbilical redness and jaundice were significantly predictors in late onset sepsis (Table 3).

**Table 3:** Clinical characteristics and positive blood culture among early onset and late onset neonatal sepsis (N 300)

Parameter (N)	Early onset sepsis ≤ 72 hrs			Late onset sepsis > 72 hrs		
	**N** **(121)**	**Culture positive n (%)**	**P value**	**N** **(179)**	**Culture positive n (%)**	**P = Value**

**Temperature**						
< 36°C (68)	31	17 (55.0)		37	26 (70.3)	
> 37.5°C (232)	90	40 (44.0)	0.214	142	66 (46.5)	0.008

**Respiratory rate**						
30-60 b/m (141)	61	22 (36.1)		80	36 (45.0)	
> 60 b/m (159)	60	35 (58.3)	0.011	99	56 (56.6)	0.82

**Chest indrawing**						
Yes (174)	69	36 (52.2)		105	60 (57.1)	
NO (126)	52	21 (40.4)	0.270	74	32 (43.8)	0.071

**Umbilical redness**						
Yes (50)	20	11 (55.0)		30	21 (70.0)	
NO (250)	101	46 (45.5)	0.298	149	71 (47.7)	0.020

**Lethargy**						
Yes (119)	55	39 (71.0)		64	50 (78.0)	
NO (143)	66	18 (27.3)	0.0001	115	42 (36.5)	0.0001

**Unable to feed**						
Yes (263)	107	57 (53.3)		156	89 (57.1)	
NO (37)	14	0 (0.0)	0.0001	23	3 (13.0)	0.0001

**Cyanosis**						
Yes (78)	36	24 (66.7)		42	27 (64.3)	
No (222)	85	33 (38.5)	0.004	137	65 (46.6)	0.041

**Jaundice**						
Yes (54)	21	13 (62.0)		33	24 (73.0)	
NO (246)	100	44 (44.0)	0.155	146	68 (46.0)	0.005

**Convulsion**						
Yes (57)	24	20 (83.3)		33	25 (76.0)	
NO (243)	97	37 (38.0)	0.0001	146	67 (46.0)	0.002

About 28.5% of neonates with positive blood culture died compared to only 8.6% of those with negative blood culture p = 0.0001. Gram negative sepsis had higher mortality than gram positive sepsis. Increased mortality was also seen with sepsis due to ESBL and MRSA isolates (Table 4). Mortality in early onset disease was 28(23.1%) compared to 29(16.1%) in late onset disease (p = 0.089). The outcome of neonates with sepsis caused by sensitive isolates was relatively good, 80.8% of neonates with sensitive isolates improved after 72 hrs of treatment compared to 2.2% of those with resistant isolates (p = 0.0001). Overall a total of 77(74%) neonates with sensitive isolates survived compared to only 15 (33.3%) of those with resistant isolates (Table 5).

**Table 4:** Factors associated with increased neonatal deaths among neonates with neonatal sepsis

Parameter	N	Death (%)	P value
**Culture**			
Positive	149	28.5	
Negative	151	8.6	0.0001

**Gram reaction**			
Positive	58	19	
Negative	91	36.3	0.0001

**ESBL**			
Positive	36	52	
Negative	55	25	0.008

**MRSA**			
Positive	9	55	
Negative	23	21	0.008

**Table 5:** Outcome of neonates with positive blood culture in relation to duration of treatment and sensitivity pattern

Duration of treatment	Improved/Discharged N (%)	Critical conditions N (%)	Died N (%)	P = Value
**24 hrs**				
Sensitive (104)	57 (54.8)	50 (45.2)	0 (0.0)	
Resistant (45)	0 (0.0)	42 (93.3)	3 (6.7)	p = 0.0001

**48 hrs**				
Sensitive (104)	73 (70.2)	15 (14.4)	16 (15.4)	
Resistant (45)	14 (31.1)	18 (40.0)	13 (28.9)	P = 0.0001

**72 hrs**				
Sensitive (104)	84 (80.8)	4 (3.8)	16 (15.4)	
Resistant (45)	1 (2.2)	24 (53.3)	20 (44.5)	p = 0.0001

**> 72 hrs**				
Sensitive (104)	77 (74.0)	-	27 (26.0)	
Resistant (45)	15 (33.3)	-	30 (66.7)	P = 0.0001

The p value indicates the different in outcome among neonates treated with sensitive and those with resistant antibiotics.

## Discussion

This study at BMC neonatal unit shows the prevalence of neonatal sepsis among neonates admitted during study period is about 39%. These findings are consistent with reports from other developing countries and those done in other tertiary hospitals in Tanzania [9-11]; but higher than those reported in developed countries [1]. The variations between developed countries and developing countries are due to high quality of life and hospital services in developed countries [1].

In this study the mortality rate of neonatal sepsis was 19% which is similar to that observed in other studies in East Africa region [11,12]; this can be explained by relative similar management practices and similar hospital services. Higher mortality rate was observed among neonates with early onset disease although the difference observed was not statistically significant. Among children suspected with neonatal sepsis using clinical criteria 49% were confirmed to have septicemia and no significant difference was observed among neonates with early or late onset sepsis. These findings are similar to those obtained in other studies in Africa which have been found to range from 25-54% [9,10,18]. When the findings are compared to the study in Uganda [11] in which proven sepsis was 37% among 293 neonates, the difference could be explained by stringent criteria used in the present study.

In the present study clinical characteristics such as inability to feed, cyanosis, lethargy and convulsion were found to be significantly associated with positive blood culture (p < 0.05) in both early and late onset neonatal sepsis, similar findings were observed in other studies [11,19]. Hypothermia, maternal fever, umbilical redness and jaundice were also found to be predictors of positive blood culture only among neonates with late onset sepsis whereas increased respiratory rate was significant predictor of positive blood in early onset disease only. These clinical signs and symptoms laid by WHO Young infants study group [6] could be used in the areas of limited facilities to predict positive blood culture and initiation of proper empirical management.

As in other studies perinatal factors which were strongly found to predict positive blood culture in both early and late onset disease were PROM and meconium stained liquor [19,20]. Of neonates with late onset disease positive blood culture rate was significantly higher in those delivered at hospital (p = 0.006). This could partly be explained by type of organisms isolated, most of the isolates in the present study were multiply resistant, possibly hospital acquired infections. Place of delivery had no influence on positive blood culture among neonates with early onset sepsis. There was no control group in the present study and only neonates with blood culture drawn were included in the analysis; this could have caused selection bias in determining the predictors of positive blood culture.

Organisms which were found significantly causing septicaemia in PROM were *Escherichia coli*, *Enterobacter spp*, *Group B streptococci *and *Klebsiella pneumoniae *(p < 0.05), this could be due to the ascending infection from perineum and vagina. As in other studies [2,20,21] these pathogens are commonly responsible for early onset disease and this was confirmed in the present study.

In contrast to other studies in developed countries [22], gram negative bacteria formed majority of the isolates in our study. *Klebsiella pneumoniae *was the commonest isolate recovered in the present study. The predominance of an organism causing septicemia in the unit can be due to selective pressure of antibiotics, this has been found to be true with neonatal septicemia due to *Klebsiella pneumoniae *[20]. In our study majority of *Klebsiella pneumoniae *were resistant to multiple antibiotics with 49% of these isolates producing extended spectrum beta-lactamases (ESBL). *Escherichia coli *were the second commonest gram negative bacteria with majority of them causing early neonatal sepsis and 45.5% were found to be ESBL producer. About 68% of *Klebsiella pneumoniae *and *Escherichia coli *isolates in the unit were resistant to gentamicin and 90% resistant to ampicillin. This poses challenge in the use of these first line drugs in the management of neonatal sepsis in our setting. In the unit the second choice of treatment is third generation cephalosporins of which more than 49% of gram negative isolates were found to be resistant. High resistance rate of third generation cephalosporins can be contributed by irrational use of these antibiotics in the unit; this can further be supported by low resistance rate of ciprofloxacin, the drug which is not commonly prescribed to neonates and there is no pharmacokinetic information to guide dosing of ciprofloxacin in infants [4,23]. About 95% of gram negative enteric bacteria were sensitive to meropenem similar findings have been reported elsewhere [23]. This drug is expensive and not available in many centers in developing countries, this makes the management of neonatal sepsis due to multi-resistant gram negative bacteria to be difficult in these countries.

*Staphylococcus aureus *was the second common isolate in this study and the leading among gram positive isolates. *Staphylococcus aureus *was significantly recovered in neonates from mothers without PROM (p < 0.05) and it was the commonest isolate in the late onset sepsis. About 28.1% of *Staphylococcus aureus *were found to be MRSA and about 91% resistant to penicillin, similar observation was made by Mugalu *et al *[11]. The significant association of late onset disease and *Staphylococcus aureus *septicaemia (p = 0.008), could partly be explained by the fact that these organisms are transferred to neonates from health care workers and relatives. In contrast to other studies [1,2], gram positive such as Group B Streptococci and *Listeria spp *were not commonly isolated in this study. Only two blood cultures were positive for group B streptococci (1.3%) and these were found in early onset sepsis. *Listeria spp *was isolated in 5 samples of which 3 were from early onset sepsis.

Different factors were found to contribute to increased mortality rate in the present study. As demonstrated in previous studies the outcome of sepsis is usually determined by duration of inflammatory response to the offending pathogens, with severe and worse outcome in the gram negative sepsis [18,24]. Factors which were significantly found to predict deaths in this study were being blood culture positive, isolation of gram negative bacteria, ESBL isolate and MRSA isolate. As in other studies gram negative sepsis has been associated with severe sepsis and increased mortality [21,23] and this was confirmed in our study. Routine report of laboratory results gram stain reaction could have significant influence in the management and outcome of the neonatal sepsis in our setting and other setting in developing countries. High mortality rate was observed among neonates infected with ESBL producing organisms in the present study [21,23]. In this study as in other few studies no significant difference was observed in mortality among neonates with early or late onset sepsis [11,23,24]. Majority of neonates with multi-resistant organisms died within 72 hrs of initiation of antimicrobials. Relative good survival was demonstrated in neonates with sensitive organisms, more than 80% of them improved after 72 hrs of treatment, this has been reported elsewhere [23,25].

Currently there is no guideline to treat neonatal sepsis due ESBL isolates in most centers in the developing countries. In this study high dose of gentamicin in combination with high dose of cefotaxime was used, and in some cases ciprofloxacin was used. Survival rate was 47% among 36 cases, survival was better with ciprofloxacin; than cefotaxime+ gentamicin, twenty neonates survived of which 13/20 (65%) received ciprofloxacin.

## Conclusion

Neonatal sepsis is common in our setting and has high mortality; convulsion, lethargy, inability to feed, cyanosis, PROM and meconium stained liquor were significantly found to predict positive blood culture in both early and late onset neonatal sepsis. Mortality and morbidity of neonatal sepsis is high in our setting and significantly contributed by positive blood culture with multi-resistant gram negative bacteria. Guidelines for management of neonatal sepsis in developing countries are needed so that morbidity and mortality of sepsis due to highly prevalent multi-resistant gram negative bacteria and MRSA can be reduced.

## Competing interests

The authors declare that they have no competing interests.

## Authors' contributions

NK participated in collecting specimens, collecting clinical data, treating neonates and processing samples, ER participated in planning, data analysis and manuscript writing, SJ participated in microbiological analysis, DM participated in manuscript writing, SEM participated in design and execution of the work including microbiological procedures, data analysis, interpretation of data and preparation of the manuscript. All authors have read and approved the final manuscript.

## Pre-publication history

The pre-publication history for this paper can be accessed here:

http://www.biomedcentral.com/1471-2431/10/39/prepub
